# Prevalence and risk factors associated with psychological distress among children in Penang, Malaysia: A stratified multistage cluster study

**DOI:** 10.1016/j.mex.2024.103069

**Published:** 2024-11-22

**Authors:** Xin Yee Foo, Nur Arzuar Abdul Rahim, Lai Kuan Lee

**Affiliations:** a Food Technology Program, School of Industrial Technology, Universiti Sains Malaysia, 11800 Gelugor, Pulau Pinang, Malaysia; b Department of Clinical Medicine, Universiti Sains Malaysia Bertam Medical Center, 13200 Kepala Batas, Pulau Pinang, Malaysia

**Keywords:** Children, Mental health, Prevalence, Psychological distress, Risk factors, Stratified multistage cluster sampling

## Abstract

Mental health is a state of mind influences one thinking, feeling and acting from inside and outside that are vital for children's normal growth and development. Psychological distress may results in serious mental health problem if left untreated. Hence, early diagnosis can largely improve the condition from being deteriorating. This study determined the prevalence of psychological distress and its associated risk factors among children in Penang, Malaysia. The study applied stratified multistage cluster sampling for the recruitment of children, and their socio-demographics background, health and lifestyle practices, and the prevalence and risk factors of psychological distress were succinctly studied. The study provides a fundamental platform for informing parents and policy makers about psychological distress, and the need to strategize potential health intervention for achieving optimum human well-being.

• Stratified multistage cluster sampling was useful to study the prevalence and risk factors of psychological distress in a children population.
• DASS-Y is robust for brief dimensional measure of depression, anxiety and stress among children.

## Graphical abstract

**Figure:**
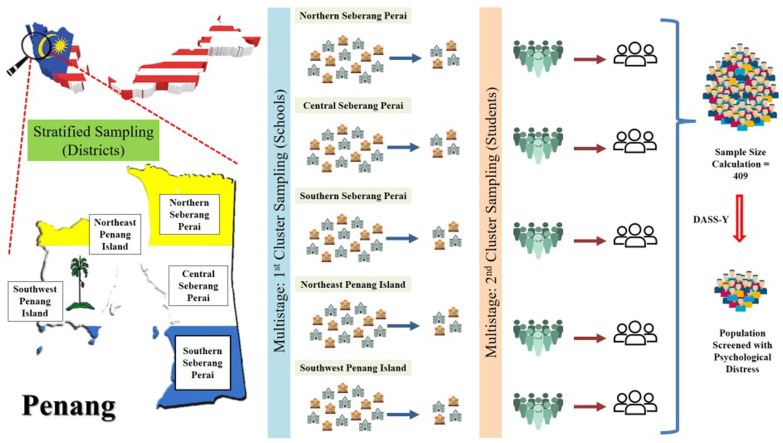

Specifications table

**Table:** 

Subject area:	Psychology
More specific subject area:	Psychological distress among children
Name of your method:	Stratified multistage cluster sampling
Name and reference of original method:	1. L.A. Latiff, E. Tajik, N. Ibrahim, A.S. Abubakar, S.S.B. Ali, Depression and Its Associated Factors among Secondary School Students in Malaysia, The Southeast Asian Journal of Tropical Medicine and Public Health 47 (1) (2016) 131–141. 2. M. Szabo, P.F. Lovibond, Development and Psychometric Properties of the DASS-Youth (DASS-Y): An Extension of the Depression Anxiety Stress Scales (DASS) to Adolescents and Children, Frontiers in Psychology 13 (2022) 766890. 3. W.W. Daniel, Biostatistics: A Foundation for Analysis in the Health Sciences, 6th ed. New York: John Wiley & Sons, 1995. 4. P.A. Lachenbruch, S.K. Lwanga, S. Lemeshow, Sample Size Determination in Health Studies: A Practical Manual, Journal of the American Statistical Association 86 (416) (1991) 1149.
Resource availability:	Not applicable

## Background

Mental health is a state of mind characterized by good behavioral adjustment, emotional well-being, cope with the ordinary demands and stresses of life, a capacity to establish constructive relationships and relative freedom from disabling and anxiety symptoms [1]. It is a vital part of children's overall health, with a complex interactive relationship combined with physical health and ability to perform well in school, society and at work. Mental health plays important roles from prenatal considerations through transitions to adulthood, which covered the whole childhood [2]. Anxiety, depression, conduct disorders, behavior disorders and attention deficit hyperactivity disorder are among the most common form of children's mental health problems [3].

The global prevalence of mental health conditions among children aged 10–19 was 1 in 7 (13 %) [4]. An epidemiological study reported by Sacco et al. [5] found that around 1 in every 5 children and adolescents (15.5 %) suffered from mental disorder. An estimation of over 11 million European children and youngsters below 20 years old are currently facing mental health problems, with a pooled prevalence of 13 % [6]. In United States, nearly 20 % of teenagers aged 3–17 experienced some kind of emotional, mental, behavioral and developmental disorders [7]. The American Psychological Association reported that an estimation of 20 million young people are being diagnosed with mental health disorder [2].

In China, 17.5 % of 17,524 individuals aged 6–16 years old were found to be having mental disorder, based on Mini-International Neuropsychiatric Interview for Children and Adolescents [8]. Another epidemiological study carried out by Zhou et al. [9] reported that about 20.3 % among 2679 children from 25 provinces in China were screened with depression. Adding to this body of knowledge, 59.0 %, 54.4 % and 24.7 % of 845 Vietnam students (10–18 years) were found to be suffering from depression, anxiety and stress, respectively [10]. In Singapore, Chodavadia et al. [11] showed that parents-reported prevalence of depression and anxiety problems was 16.2 %.

In Malaysia, according to the National Health and Morbidity Survey, the prevalence of children's mental health problems were 13.0 % in 1996, 19.4 % in 2006, 20.0 % in 2011, 12.1 % in 2015 and 7.9 % in 2019 [12]. Sahril and coworkers [12] conducted a study in urban and rural areas in Malaysia, reported that 11.1 % of pediatrics population aged 5–15 years old were suffering from mental health issues. Very recently, Ang [13] revealed that 58 % of the participants aged between 8 and 17, recruited from the Federal Territory of Kuala Lumpur, reported anxiety symptoms.

Psychological distress exerts detrimental effects on children's ability to fulfill their potential, perform well in social situation, at school and home [14]. Markedly, delays will appear in children's developing age-appropriate social adroitness, psychic, mood, conduct regulation, thought and their mindset. Consequently, their capability to live a productive life will be restricted and their academic fulfilment will be affected. Cases left untreated may interfere and prevent children from developing healthily and thus causing even worse situation extended into adulthood [15].

A number of researches evaluated the risk factors associated with psychological distress among children. Common factors including biology and genetics [2], ethnicity, age, female gender [16], parental stressors in daily life [17], parental unemployment [18,19] and stressful life events [20,21]. Changes in family environment such as family conflicts [22] and single parents [12,23] tended to contribute to children's mental health problems. Children who found to be having parents with either mental health problems [12,[24], [25], [26]] or chronic health problems [20] developed psychological distress. Furthermore, lower socioeconomic status [12], having low parental supervision, felt lonely, and being bullied were identified as the significant predictors of adolescents’ mental health problems [23,27]. Additionally, levels of physical activity or exercise significantly affected the likelihood of exhibiting mental health problems [28].

**Fig. 1:**
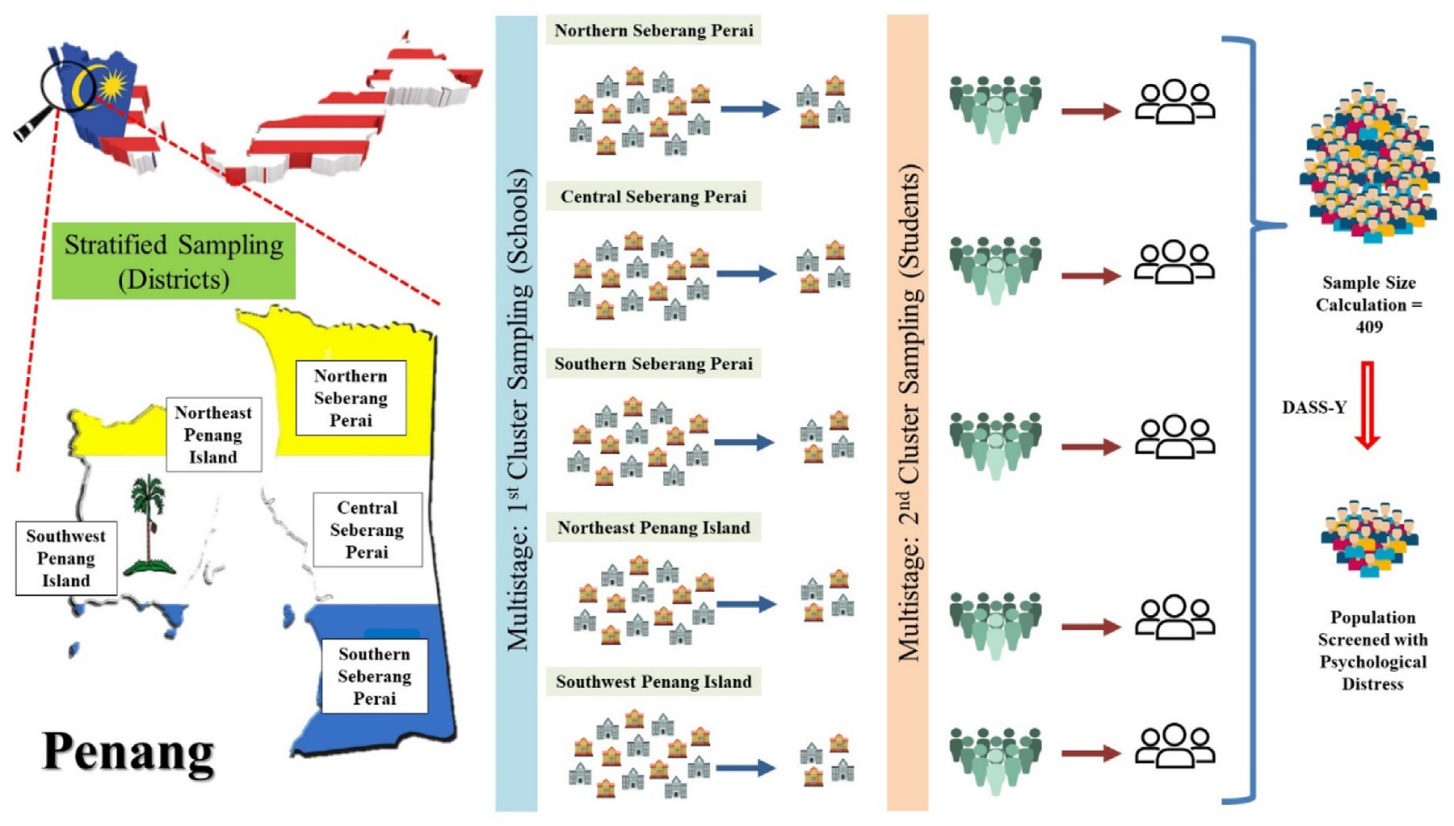
Stratified multistage cluster sampling.

The present study adopted the stratified multistage cluster sampling method to determine the prevalence and risk factors of psychological distress among children (Fig. 1). It is comparatively cost and time-effective. DASS-Youth Version (DASS-Y), a newly developed, narrower and more specific age group (7–18) appropriate, covering the 3 most common psychological distress problems, validated and charge-free instrument was used to collect huge amount of useful data in short period [29]. Its existence filled up the gap in current repertoire of instruments in assessing youth's negative emotion. The study was conducted in a non-formal, fun, interactive and non-stressful way to reduce the avoidance feeling of subjects.

## Method details

**Fig. 2:**
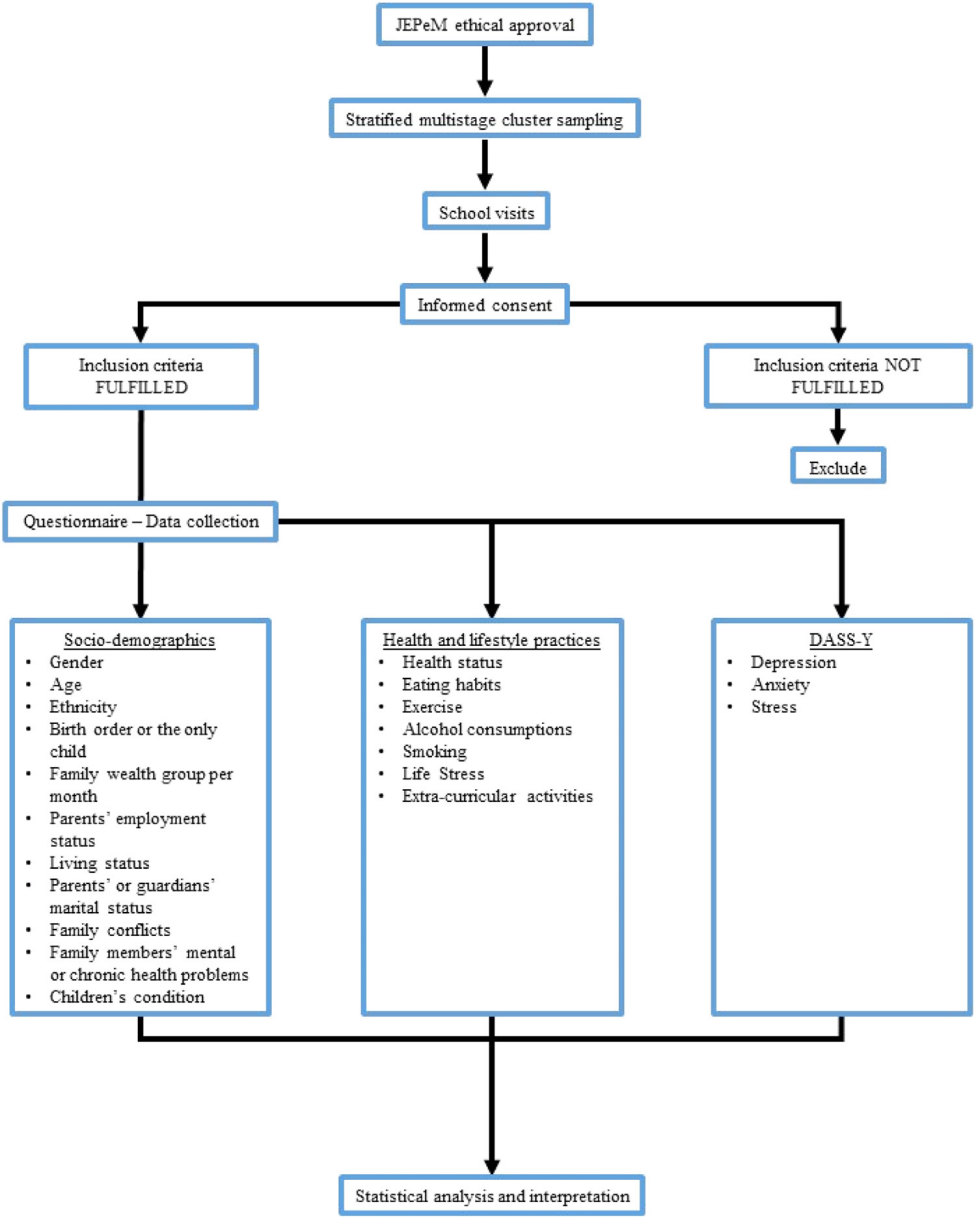
Flow chart of the method.

Fig. 2 illustrates the overall flow of the study.

### Sample and design

Participants involved in the present study were children aged 10–11 years covering all races (Malay, Chinese and Indian), and studied in either National Primary School (SK), Chinese National Primary School (SJK(C)) or Tamil National Primary School (SJK(T)) in Penang state, Malaysia. In stratified sampling, the geographical division followed the official municipality districts of Penang state, listed as Northeast Penang Island, Southwest Penang Island, Northern Seberang Perai, Central Seberang Perai and Southern Seberang Perai. Volunteers were then recruited through multistage cluster sampling method as suggested by Lachenbruch et al. [30]. A random selection of schools from each district was adopted based on a random number table [31,32], following a standard races ratio of 6:3:1 in Malaysia, for Malay, Chinese and Indian, respectively. The second stage of cluster sampling stratified students in the age clusters of 10 and 11 years old. Participations were fully volunteered and expressed their willingness to join the study. They were invited face to face with the help of school administrative staffs, as well as using informational sheets and word-of-mouth invitation. Subjects were excluded if they met any of the exclusion criteria (Table 1). The study recruitment and enrolment began in April 2024, and completed by June 2024.

**Table 1:** Inclusion and exclusion criteria for study population.

Inclusion criteria	Exclusion criteria
1. Male or female 2. Chronological age: 10–11 years old 3. Students from SK, SJK (C) or SJK (T)	1. Children with documented mental retardation or psychiatric disorders (e.g., autism, attention deficit hyperactivity disorder, bipolar disorder, major depression, schizophrenia, behavioral and emotional disorders, dissociation and dissociative disorders, eating disorders, obsessive compulsive disorder, paranoia, post-traumatic stress disorder, neurodevelopmental disorders, borderline personality disorders, panic disorders, personality disorders, as documented by medical report) 2. Those having symptoms due to direct psychological effects of substance (medication use, information provided by the parents or guardians) or a general medical condition

### Sample size and power calculation

A standard formula specifically designed to be used for conducting prevalence study was used [33] to calculate the required sample size. Results from Latiff and colleagues [32] are adopted to determine the sample size for this study.

$$n=\frac{Z^{2}P\;\left(1-P\right)}{\;d^{2}}$$

$$n=\frac{{\left(1.96\right)}^{2}\left(0.332\right)\left(1-0.332\right)}{\;{\left(0.05\right)}^{2}}$$

n=341subjects

where n = sample size, P = Expected prevalence or proportion, Z = Z statistic for a level of confidence (95 %) and 80 % power, and d = Precision. With the consideration of 20 % dropout rate, the required sample size was 409 subjects.

### Study instrument

A semi-quantitative questionnaire was used to gather socio-demographic details, included gender, age (10 or 11 years), ethnics (Malay, Chinese or Indian), birth order, family wealth group (in Malaysian Ringgit: B40 ≤ RM4850; M40 = RM4850-RM10959 and T20 ≥ RM10959), parents’ employment status (working or not working/housewife), living status (defined as staying with family, with relatives or others), parents’ or guardians’ marital status (defined as married, remarried, divorced or widowed). Information about the occurrence of family conflicts, and family members with chronic or mental health problems were obtained. Assessment on children's condition included parental stressors in daily life, life threatening illness/accident/incident/attack, felt helpless, felt very uncomfortable, being bullied/attacked/harmed, immediate family member/partner/very close friend passed away due to accident/being killed/suicided, present when another person was seriously injured/physically assaulted, been in any situation that was extremely frightening/horrifying and whether they are school representatives for sports.

In addition, assessment on health and lifestyle practices required the participant to declare any medical prescriptions, and current and past medical history. Self-reported daily eating habits, the frequency and duration of exercise practices (defined as: never, 1–3 times per week, 4–7 times per week and >7 times per week; duration: 10–20 min, 20–30 min, 30–40 min and >40 min) were obtained. Assessment that determined whether they consume alcohol, with the responses recorded as drinking frequencies (never, less than once a month, all month, all weeks and all days), types of alcoholic drinks (beer, wine, spirits, and mixers), age of first tried and drunk experiences (no, 1 time, 2–3 times, 4–10 times and >10 times) were included. Subjects were also asked to response whether they are a smoker, ever smoke and any smoker among the family members. Life stress level was indicated as Likert scale 6 (not at all stressful) to 1 (very stressful). Participation in any extra-curricular activities were self-reported, and participants answered the type of activities, positions hold and participation levels.

Psychological distress among children was screened using the Depression, Anxiety and Stress Scales – Youth version (DASS-Y). DASS-Y provides a psychometrically sound brief dimensional measure and can possibly identifying a core set of symptoms which define depression, anxiety and stress in children and adolescents. It is empirically based and giving scores graded in severity that reflects the distribution of symptom levels among children [34]. The English original version of DASS-Y had acceptable internal consistency, with the Cronbach's alpha coefficient of 0.89 (depression), 0.84 (anxiety) and 0.84 (stress) based on ratings of 2121 Australian children and adolescents aged 7–18 [34]. The instrument consists of 21 items, and all items are categorized into 3 domains, where each domain comprising 7 items (Table 2). These domains are depression, anxiety and stress. The screening was completed by participants on their own with available responses based on 4-points Likert scale ‘0 – Not True’, ‘1 – A Little True’, ‘ 2 – Fairly True’ and ‘3 – Very True’. Each domain has a score range from 0–21 that can be subdivided into different graded severity level: normal, mild, moderate, severe or extremely severe. Scores under each of the domain can be summed up to obtain a total score meant for the level of psychological distress: 0–23 (Normal), 24–29 (Mild), 30–39 (Moderate), 40–46 (Severe) and >46 (Extremely Severe) [35].

**Table 2:** Content of questionnaire.

Section A Socio-demographics	Section B Health and Lifestyle Practices		Section C DASS-Y
1) Gender 2) Age 3) Ethnicity 4) Siblings: - Birth order - Are you the only child? 5) Family wealth group (per month) 6) Parent's occupational status 7) Living status 8) Parents’ or guardians’ marital status 9) Family members with chronic health problems? 10) Family members with mental health problems? 11) Family conflicts: - Extramarital affairs - Financial difficulties - Communication failure or poor family communication - Parenting issues - Arguments - Parent-child power struggles - Different cultures and viewpoints - Sibling rivalry - Disagreements over financials and occupations - Conflict regarding child-rearing or discipline techniques - Measures and principals - War with parents-in-law 12) Do you feel any parental stressors in daily life? 13) Have you ever had a life-threatening illness/accident/incident/attack that harmed you? 14) Had an immediate family member, partner or very close friend passed away because of accident, being killed or suicided? 15) Anytime in your life you felt helpless? 16) Anytime in your life you felt very uncomfortable? 17) Have you ever had an experience with others bullying or attacking you that harmed you? 18) Have you ever been present when another person was seriously injured or physically assaulted (attacked)? 19) Have you ever been in any situation that was extremely frightening or horrifying? 20) Are you a school athletes or school representatives for sports? (e.g., basketball, football, netball, dodgeball, hiking, badminton, baseball, swimming, cycling, volley ball, bowling, gymnastics, golf, track and field, martial arts, tennis, taekwondo, dancing, softball, squash, rugby, ping pong, hockey, kayaking etc.)	I. Health status 1) Are you currently suffering from any medical condition that affects your health? 2) Are you currently on any medication? 3) Have you ever had an operation? 4) Are you currently or have you ever been registered disabled? 5) Suffered from or are currently affected by: a. Allergies to: - Pollen - Dust - Dust mites - Mould - Insect stings - Latex - Seafood - Medication - Cockroach - Overactive immune system - Cereals - Eggs - Legumes/nuts/seeds/beans - Dairy products - Chemicals b. Asthma c. Ear trouble d. Eye trouble e. Fainting or blackouts f. Heart trouble g. Hepatitis h. Recurring headaches i. Mental illness j. Stomach trouble k. Fear of heights l. Body cramps m. Eczema (dry, itchy or inflamed skin) II. Eating habits 1) I drink between 4 and 8 glasses of water a day 2) I eat sweets, ice cream, cakes more than 2 times a week 3) I eat less than 2 rations of fruits per day 4) I eat less than 1 ration of vegetables per day 5) I eat 2 or more rations of meat products per day 6) I consume less than 3 servings of milk or derivatives per day 7) I eat 2 or more rations of pastries per day 8) I eat salty snacks (crisps, chips, snacks…) or fast food 2 or more rations per week 9) In the last 12 months, I have been on a weight loss diet 10) I go to school without breakfast 11) I often skip meals 12) I like to eat nuts 13) I drink coffee	III. Exercise 1) Do you exercise (at least once in a week)? 2) If yes, a. Light exercise, such as the following: - light gardening and light housework (e.g., dusting, sweeping, vacuuming) - leisurely walking (e.g., walking your dog) - bowling, fishing, carpentry, playing a musical instrument - volunteer work b. Moderate exercise, such as the following: - brisk walking - bicycling, skating, swimming, curling, kayaking - gardening (e.g., raking, weeding, digging, spading) - dancing, tai chi, taekwondo, martial arts, gymnastics, golf or moderate exercise classes c. Vigorous exercise, such as the following: - running, track and field, skiing, lap swimming, hiking, aerobics - heavy yard work - weight training - soccer, basketball, ping pong, hockey, football, netball, dodgeball, tennis, baseball, volley ball, softball, badminton, squash, rugby or other league sports IV. Alcohol consumptions 1) How often do you currently drink alcoholic beverages? 2) At what age did you remember having your first drink of any alcoholic beverage? 3) Have you ever drank so much that you got drunk? V. Smoking 1) Are you a smoker? 2) If no, did you ever smoke? 3) Any of your family member is currently smoking? VI. Life stress VII. Extra-curricular activities (e.g., competition, uniform body, club, organization, association, agency, sport, event, talent etc.)	Depression 3 - I did not enjoy anything. 5 - I hated my life. 10 - There was nothing nice I could look forward to. 13 - I could not stop feeling sad. 16 - I hated myself. 17 - I felt like I was no good. 21 - I felt that life was terrible. Anxiety 2 - I felt dizzy, like I was about to faint. 4 - I had trouble breathing (e.g., fast breathing), even though I wasn't exercising and I was not sick. 7 - My hands felt shaky. 9 - I felt terrified. 15 - I felt like I was about to panic. 19 - I could feel my heart beating really fast, even though I hadn't done any hard exercise. 20 - I felt scared for no good reason. Stress 1 - I got upset about little things. 6 - I found myself over-reacting to situations. 8 - I was stressing about lots of things. 11 - I was easily irritated. 12 - I found it difficult to relax. 14 - I got annoyed when people interrupted me. 18 - I was easily annoyed.

### Variables and risk factors

Children were considered having psychological distress if the DASS-Y score was beyond 23, and this served as the dependent variable. Independent variables included socio-demography, health and lifestyle practices (possible predictors of psychological distress) (Table 2).

### Statistical analysis

All data were analyzed using the Statistical Package for Social Sciences (version 27.0.1; SPSS Inc., Chicago, IL, United States) software. Using descriptive statistics, categorical data (socio-demography, health and lifestyle practices) were presented as frequencies and percentages. Continuous data were reported in ranges, median and means with standard deviations. Prevalence of psychological distress was presented in percentage. Pearson chi-square test was carried out to compare the differences in the DASS-Y scores according to various categorical variables, together with 95 % confidence interval (CI), as appropriate. Stepwise binary logistic regression analysis was used to perform variable selection and simultaneously examined the association between relative predictors and psychological distress according to the crude odds ratio (ORs) produced, where the response variables were set in dichotomous form. All statistical tests were 2-tailed at an alpha level of 0.05. *p*-value below 0.05 was considered as statistically significant.

## Method validation

Data analysis for the method is currently underway.

## Limitations

The successful implementation of this method hinges on the schools, parents/guardians and subjects’ activeness and willingness to take part in the study. Lower level of awareness, personal data privacy and time availability were their main concerns. It is good to include subjects from wider age groups to have a better image on the trend of psychological distress among children.

## Ethics statements

The study has obtained ethical approval from the Human Research Ethics Committee of Universiti Sains Malaysia (No.: USM/JEPeM/PP/23070575). The permission to enroll the school-going kids as subjects was granted from Ministry of Education (MoE). Data collection was conducted according to the Code of Ethics of the World Medical Association (Declaration of Helsinki) for experiments involving humans. Informed consent for participation and publication consent were signed by all subjects.

## Funding

The work was supported by Sacha Inchi Manufacturing Sdn Bhd (R417).

## Supplementary material *and/or* additional information [OPTIONAL]

Not Applicable.

## CRediT authorship contribution statement

**Xin Yee Foo:** Methodology, Formal analysis, Investigation, Data curation, Writing – original draft. **Nur Arzuar Abdul Rahim:** Supervision, Project administration. **Lai Kuan Lee:** Supervision, Conceptualization, Validation, Project administration, Funding acquisition, Resources, Writing – review & editing.

## Declaration of competing interest

The authors declare that they have no known competing financial interests or personal relationships that could have appeared to influence the work reported in this paper.

## Data Availability

No data was used for the research described in the article.
